# Stress exposure prior to diagnosis and clinical characteristics in systemic lupus erythematosus: a cross-sectional study

**DOI:** 10.1007/s00296-026-06183-4

**Published:** 2026-06-13

**Authors:** Eleni Papachristodoulou, Loukas Kakoullis, Costas A. Christophi, Chris T. Derk, Andreas Chatzittofis, Panagiotis Alexopoulos, Menelaos Karanikolas, Dimitrios Velissaris, Konstantinos Parperis

**Affiliations:** 1 Department of Medicine, Mount Auburn Hospital, 330 Mount Auburn St., Cambridge, MA 02138 USA; 2 Harvard Medical School, Boston, MA USA; 3 Department of Medicine, University of Patras School of Health Sciences, Patras, Greece; 4 Department of Medicine, Massachusetts General Hospital, Boston, MA USA; 5 Cyprus International Institute for Environmental and Public Health, School of Health Sciences, Cyprus University of Technology, Limassol, Cyprus; 6 Department of Rehabilitation Sciences, School of Health Sciences, Cyprus University of Technology, Limassol, Cyprus; 7 Division of Rheumatology, University of Pennsylvania, Philadelphia, PA USA; 8 Department of Psychiatry, University of Cyprus Medical School, Nicosia, Cyprus; 9 Mental Health Services, Faculty of Medicine, School of Health Sciences, Patras University Hospital, University of Patras, Patras, Greece; 10 Department of Anesthesiology, Washington University School of Medicine, St. Louis, MO USA; 11 Department of Internal Medicine, University General Hospital of Patras, Patras, Greece; 12 Department of Internal Medicine, Division of Rheumatology, University of Cyprus Medical School, Nicosia, Cyprus

**Keywords:** Lupus erythematosus, systemic, Stress, psychological, Patient-reported outcome measures, Antiphospholipid syndrome, Depression, Comorbidity

## Abstract

Psychological stress has been linked to the onset and course of autoimmune diseases. However, limited data exist regarding the relationship between pre-diagnosis stress exposure and subsequent disease course in systemic lupus erythematosus (SLE). We evaluated the association between psychological stress prior to SLE diagnosis and subsequent disease characteristics. We conducted a cross-sectional study of adults with SLE treated at a tertiary hospital between November 2019 and December 2022. Stress exposure during the two years preceding SLE diagnosis was assessed using the Holmes-Rahe Social Readjustment Rating Scale (life change units, LCU), and categorized as low (< 150), intermediate (150–299), or high (≥ 300). Data on clinical manifestations, comorbidities, disease activity patterns, patient-reported outcomes, and organ damage were collected. Associations between stress levels and outcomes were analyzed using logistic regression models. A total of 140 patients were included (90.7% female; median age at diagnosis 35.5 years). Moderate-to-high stress exposure (LCU ≥ 150) was reported by 46% of patients. Compared with low stress exposure (< 150 LCU), high stress exposure (≥ 300 LCU) was associated with significantly higher rates of depression (62.5% vs. 28.0%; *p* = 0.002), hypertension (54.2% vs. 24.0%, *p* = 0.006), and obesity (12.5% vs. 0.0%, *p* = 0.013), as well as greater fatigue burden (median 68.7 vs. 46.9, *p* = 0.005). High stress exposure was associated with antiphospholipid syndrome (OR 6.3, 95% CI 1.4–28.8), depression (OR 4.3, 95% CI 1.6–11.3), and a lower likelihood of long quiescent disease course (OR 0.22, 95% CI 0.07–0.7). Patients with higher stress exposure more frequently believed that stress contributed to both disease onset (100% vs. 71.7%, *p* = 0.006) and flares (100% vs. 69%, *p* = 0.006). Elevated stress levels preceding SLE diagnosis are associated with comorbidities and less favorable disease patterns. These findings underscore the clinical relevance of psychological stress in SLE and support the need for prospective studies examining its relationship with disease course.

## Introduction

Systemic Lupus Erythematosus (SLE) is a chronic, multisystemic autoimmune disease. It is characterized by immune dysregulation, leading to the production of autoantibodies, immune complexes, autoreactive T cells, and pro-inflammatory cytokines [1, 2].

There is increasing evidence that psychological stress is associated with both the onset and course of autoimmune diseases [3–5]. Stress is defined as the physical or emotional response experienced when an individual encounters changes or demands in their life that challenge their adaptive capacity. While stress is a normal reaction, chronic stress can lead to or exacerbate various health problems [5, 6]. The Holmes-Rahe stress assessment scale is a tool developed to study the impact of stress on disease, incorporating 43 distinct life events occurring in the preceding year. The cumulative score corresponds to the risk of developing stress-related illness in the subsequent two years [7].

Stress has been associated with several autoimmune diseases. Among women with rheumatoid arthritis, stress often increased in the year before symptoms began, and 55% of patients linked onset to a specific stressful event [8]. Similarly, individuals with psoriatic arthritis reported more early-life stressful events than those with other dermatological conditions [9]. In SLE, patients frequently report that stress precedes disease flares [10]. Additionally, research indicates that individuals with post-traumatic stress disorder (PTSD) have two to three times the risk of developing SLE compared to those without PTSD [11]. Interpersonal relationship problems have also been associated with increasing corticosteroid doses in SLE [12]. Studies in rodent lupus nephritis models indicate psychosocial stress may worsen kidney damage [13].

Although previous research has identified an association between stress and the clinical progression of SLE [14], this relationship has not been well quantified. This study aims to measure stress levels in patients during the two years prior to SLE diagnosis and to assess their correlation with patterns of disease activity, clinical manifestations, comorbidities, and patient-reported outcomes.

## Materials and methods

This cross-sectional study was conducted and reported in accordance with the Strengthening the Reporting of Observational Studies in Epidemiology (STROBE) guidelines [15]. The study population consisted of patients with a diagnosis of SLE presenting to the division of Rheumatology of the General Hospital of Nicosia, Cyprus between November 2019 and December 2022. Patients were eligible for inclusion if they met the American College of Rheumatology (ACR) and/or Systemic Lupus International Collaborating Clinics (SLICC)-2012 SLE classification criteria [16]. Only patients diagnosed after the age of 18 were included in the study. The study was approved by the Cyprus National Bioethics Committee (protocol number EEBK/2019/03; approval date March 4th, 2019). Written informed consent was obtained from all participants prior to inclusion. The study was conducted in accordance with the Declaration of Helsinki and its later amendments.

Data collected included patient demographics, past medical history including manifestations and complications of SLE, laboratory parameters, markers of SLE disease activity, and disease activity patterns (relapsing-remitting, chronic active, and long quiescent). Disease activity patterns were categorized based on overall clinical course, as assessed by physician evaluation and patient-reported disease history, using a previously described classification in the literature [17] as a conceptual framework.

Data included self-rated health, Lupus Quality of Life questionnaire (LupusQol), patient health questionnaire (PHQ)-9, physician global assessment (PhGA), patient global assessment (PGA), Safety of Estrogens in Lupus Erythematosus National Assessment-Systemic Lupus Erythematosus Disease Activity Index (SELENA-SLEDAI) and the SLICC/ACR Damage Index (SDI). A detailed description of these scales can be found elsewhere [18–20]. Depression was defined as having a PHQ-9 score of 10 or above [21]. Organ damage was defined as an SDI score ≥ 1 and active disease as SELENA-SLEDAI ≥ 4 [20].

Stress levels were quantified using the social readjustment rating scale, known as the Holmes-Rahe scale, named after the psychiatrists who developed this tool in 1967 [7]. The scale comprises 43 items assessing life events that require varying levels of social readjustment, ranging from minor incidents, such as traffic violations, to significant events, such as the death of a spouse [22]. Each event is assigned a “Life Change Unit” (LCU) score, with a cumulative score of 300 or higher indicating an elevated risk of illness over the subsequent two years; conversely, a score of 150 or lower indicates a lower risk of illness over a two-year period [22, 23].

In the present study, patients reported whether they had experienced any of the 43 events in the two years preceding their diagnosis of SLE. The scale was applied retrospectively to capture cumulative exposure to major life events in the two years preceding diagnosis, consistent with its original conceptual framework for estimating subsequent illness risk. Patients were divided into the high stress group if they had an LCU score of ≥ 300, the low stress group if they had an LCU score of < 150 and the intermediate stress group if they had an LCU score of between 150 and 299. The Holmes-Rahe Social Readjustment Rating Scale was selected because it captures exposure to defined life events rather than perceived stress, allowing for a standardized quantification of cumulative stress exposure prior to diagnosis. While it does not account for individual coping mechanisms or chronic stressors, it has been widely used in medical populations and provides a pragmatic framework for assessing stress-related illness risk [6]. Patients were also assessed for their belief of stress impact on disease flares and disease onset. The questions asked were “Do you think stress is associated with disease onset?” and “Do you think stress causes flares?”.

Statistical analyses were performed using SAS software, version 9.4. Summary statistics for demographic and clinical parameters were presented for the entire cohort and stratified by stress level group. Continuous variables were reported as mean ± SD or median (Q1, Q3), based on the normality of the distribution; group comparisons were conducted using the ANOVA or the Kruskal-Wallis tests, as appropriate. Categorical variables were reported as frequencies (%) and compared across groups using the χ² test or the Fisher’s exact test, as appropriate. Variables were summarized across all stress groups, and primary comparisons were performed between the high stress (≥ 300 LCU) and low stress (< 150 LCU) groups. Logistic regression models were used to assess associations between stress exposure and clinical outcomes. High stress exposure (≥ 300 LCU) was used as the primary independent variable, with low stress exposure (< 150 LCU) as the reference group. Separate models were constructed for each outcome, including antiphospholipid syndrome, depression, disease activity measures (SELENA-SLEDAI), disease activity patterns, and patient-reported outcomes. These outcomes were selected a priori based on their clinical relevance and representation of disease manifestations and comorbidities in SLE, and were further supported by univariate analyses. Adjusted models included age at diagnosis and gender as covariates. Given the sample size, additional covariates were not included to avoid model overfitting and instability of estimates. In light of the exploratory nature of the study and sample size constraints, no formal a priori power calculation was performed. All tests were two-tailed, and a p-value < 0.05 was considered statistically significant.

## Results

### Demographics

In total, 140 patients with SLE were included in the study (Table 1). Of these, 127 (90.7%) were female, with a median age at diagnosis of 35.5 years [interquartile range (IQR) 27–47]. The median time from symptom onset to diagnosis was 1 year (IQR 0–3), and the median duration of disease at the time of study entry was 8.5 years (IQR 2–18). The most common clinical manifestations and associated conditions were lupus nephritis (26, 18.6%), cutaneous SLE (11, 7.9%), Sjögren’s syndrome (10, 7.1%), antiphospholipid syndrome (9, 6.4%), mixed connective tissue disease (3, 2.1%), and rheumatoid arthritis (2, 1.4%). Most patients (76, 54.3%) were diagnosed with SLE without any accompanying overlapping syndromes.

**Table 1:** Baseline demographic and clinical characteristics of patients with systemic lupus erythematosus stratified by stress level

Characteristic	Overall		Holmes Rahe Scale < 150		Holmes Rahe Scale 150–300		Holmes Rahe Scale ≥ 300		p-value*
Demographics	N^a^	Mean ± SD or n (%)	N	Mean ± SD or n (%)	N	Mean ± SD or n (%)	N	Mean ± SD or n (%)	
Age at diagnosis (years)	140	38.5 ± 13.9	75	38.0 ± 14.5	41	39.7 ± 14.7	24	38.3 ± 11.0	0.91
Female (%)	140	127 (91%)	75	64 (85%)	41	39 (95%)	24	24 (100%)	0.06
BMI (kg/m^2^)	136	24.5 ± 4.6	74	24.0 ± 3.8	38	24.6 ± 4.2	24	25.8 ± 7.0	0.23
Ever smoker (%)	139	58 (42%)	74	33 (45%)	41	18 (44%)	24	7 (29%)	0.18
Marital Status (% Married)	139	98 (71%)	75	54 (72%)	40	29 (73%)	24	15 (63%)	0.38
Pregnancy (% Ever)	127	110 (87%)	64	56 (88%)	39	34 (87%)	24	20 (83%)	0.73
*Clinical characteristics*									
Antiphospholipid syndrome (%)	140	9 (6%)	75	3 (4%)	41	1 (2%)	24	5 (21%)	**0.019**
Cutaneous manifestations (%)	140	115 (82%)	75	58 (77%)	41	34 (83%)	24	23 (96%)	0.06
Renal manifestations (%)	140	35 (25%)	75	15 (20%)	41	15 (37%)	24	5 (21%)	1.0
Ophthalmologic manifestations (%)	140	52 (37%)	75	25 (33%)	41	14 (34%)	24	13 (54%)	0.07
Gastroenterological manifestations (%)	140	64 (46%)	75	30 (40%)	41	20 (49%)	24	14 (58%)	0.12
Cardiac manifestations (%)	140	14 (10%)	75	5 (7%)	41	6 (15%)	24	3 (13%)	0.40
Hematologic manifestations (%)	140	80 (57%)	75	40 (53%)	41	27 (66%)	24	13 (54%)	0.94
Vascular manifestations (%)	140	75 (54%)	75	44 (59%)	41	19 (46%)	24	12 (50%)	0.46
Musculoskeletal manifestations (%)	140	116 (83%)	75	60 (80%)	41	34 (83%)	24	22 (92%)	0.23
Cutaneous lupus erythematosus (%)	140	11 (8%)	75	7 (9%)	41	3 (7%)	24	1 (4%)	0.68
Pulmonary manifestations (%)	140	27 (19%)	75	13 (17%)	41	8 (20%)	24	6 (25%)	0.39
Neuropsychiatric manifestations (%)	140	45 (32%)	75	24 (32%)	41	13 (32%)	24	8 (33%)	0.90
*Comorbid conditions*									
Anxiety (%)	140	13 (9%)	75	8 (11%)	41	2 (5%)	24	3 (13%)	0.73
Cerebrovascular disease (%)	140	5 (4%)	75	2 (3%)	41	2 (5%)	24	1 (4%)	0.57
Depression (%)	138	50 (36%)	75	21 (28%)	39	14 (36%)	24	15 (63%)	**0.002**
Diabetes mellitus (%)	140	5 (4%)	75	2 (3%)	41	1 (2%)	24	2 (8%)	0.25
Hypertension (%)	140	42 (30%)	75	18 (24%)	41	11 (27%)	24	13 (54%)	**0.006**
Ischemic heart disease (%)	140	9 (6%)	75	4 (5%)	41	2 (5%)	24	3 (13%)	0.36
Obesity (%)	140	3 (2%)	75	0	41	0	24	3 (13%)	**0.013**
History of preeclampsia, eclampsia (%)	116	8 (7%)	58	2 (3%)	36	4 (11%)	22	2 (9%)	0.30
Miscarriages, ever (%)	111	31 (28%)	54	13 (24%)	34	9 (26%)	23	9 (39%)	0.18

*S.D* Standard deviation, *BMI* Body mass index, *SLE* Systemic lupus erythematosus

^a^ Ns vary due to missing data

*p-value: Comparisons performed between Holmes–Rahe < 150 and ≥ 300 groups. Bold values indicate statistically significant results (p < 0.05)

### Exposure to stressful life events

Regarding the levels of exposure to stressful life events in the two years prior to diagnosis, 75 patients (54%) had low (LCU < 150), 41 patients (29%) had intermediate (LCU between 150 and 299) and 24 patients (17%) had high levels of exposure to stressful life events (LCU ≥ 300). When comparing patients in the high stress group to patients in the low stress group, there was no significant difference in gender distribution (female gender: 100% vs. 85%, *p* = 0.06) or median age (39 vs. 34 years, *p* = 0.91). The most common stressful events experienced by the patients were major changes in sleeping habits (40, 28.6%), financial state (35, 25%), working hours or working conditions (33, 23.6%), death of a close family member (30, 21.4%), and major change in eating habits (29, 20.7%). A complete list of the frequency of each stressful event is presented in Table 2.

**Table 2:** Frequency of stressful life events included in the Holmes-Rahe scale [7], within two years prior to the diagnosis of systemic lupus erythematosus

Stressful life event	Life change units associated with event	*N* (%)
Death of a spouse	100	1 (0.7%)
Divorce	73	8 (5.7%)
Marital separation	65	11 (7.9%)
Detention in jail	63	2 (1.4%)
Death of a close family member	63	30 (21.4%)
Major personal injury or illness	53	24 (17.1%)
Marriage	50	13 (9.3%)
Being fired at work	47	4 (2.9%)
Marital reconciliation	45	2 (1.4%)
Retirement	45	2 (1.4%)
Major change in health or behavior of family member	44	28 (20%)
Pregnancy	40	10 (7.1%)
Sexual difficulties	39	20 (14.3%)
Gaining a new family member	39	15 (10.7%)
Major business readjustment	39	10 (7.1%)
Major change in financial state	38	35 (25%)
Death of a close friend	37	4 (2.9%)
Changing to a different line of work	36	25 (17.9%)
Major change in the frequency of arguments with spouse	35	18 (12.9%)
Taking on a major mortgage	32	13 (9.3%)
Foreclosure on a mortgage or loan	30	5 (3.6%)
Major change in responsibilities at work	29	26 (18.6%)
Child leaving home	29	17 (12.1%)
In-law trouble	29	16 (11.4%)
Outstanding personal achievement	28	18 (12.9%)
Spouse beginning or ceasing work outside the home	26	13 (9.3%)
Beginning or ceasing of formal schooling	26	16 (11.4%)
Major change in living conditions	25	28 (20%)
Revision of personal habits	24	11 (7.9%)
Trouble with boss	23	12 (8.6%)
Major change in working hours or conditions	20	33 (23.6%)
Change in residence	20	26 (18.6%)
Changing to a new school	20	7 (5%)
Major change in usual type or amount of recreation	19	26 (18.6%)
Major change in church activity	19	15 (10.7%)
Major change in social activities	18	28 (20%)
Taking on a loan (car, tv, etc.)	17	7 (5%)
Major change in sleeping habits	16	40 (28.6%)
Major change in number of family get-togethers	15	28 (20%)
Major change in eating habits	15	29 (20.7%)
Vacations	13	18 (12.9%)
Major holiday	12	13 (9.3%)
Minor violation of law	11	5 (3.6%)

Events are listed in descending order of life change units assigned in the Holmes–Rahe Social Readjustment Rating Scale

*SLE* Systemic lupus erythematosus

### Association of exposure to stressful life events to clinical characteristics and comorbidities

Compared with patients in the low stress group, those in the high stress group did not differ in measures of disease activity or cumulative damage at the time of study enrollment. Specifically, no differences were observed in SELENA-SLEDAI scores, SLICC/ACR Damage Index, PGA, self-rated health, or PhGA (Table 3). Patients with high stress levels were more likely to be obese (12.5% vs. 0.0%, *p* = 0.013), have hypertension (54.2% vs. 24.0%, *p* = 0.006), and depression (62.5% vs. 28%, *p* = 0.002). They were also more likely to score lower on the LupusQol set of questions pertaining to fatigue (median score 46.9 vs. 68.7, *p* = 0.005) and body image (median score 65.6 vs. 91.6, *p* = 0.021), suggesting that fatigue and body image had a greater impact on their lives (Table 3).

**Table 3:** Patient-reported outcomes, quality of life, disease activity, and health status in patients with systemic lupus erythematosus stratified by stress exposure level

	Total		Holmes Rahe Scale < 150		Holmes Rahe Scale 150–300		Holmes Rahe Scale ≥ 300		*p*-value*
	*N*	Median [Q1, Q3] or *n* (%)	*N*	Median [Q1, Q3] or *n* (%)	*N*	Median [Q1, Q3] or *n* (%)	*N*	Median [Q1, Q3] or *n* (%)	
**PHQ 9**	138		75		39		24		**0.008**
% Minimal depression 0–4		47 (34%)		30 (40%)		14 (36%)		3 (12%)	
% Mild depression 5–9		41 (30%)		24 (32%)		11 (28%)		6 (25%)	
% Moderate depression 10–14		35 (25%)		13 (17%)		12 (31%)		10 (42%)	
%Moderately severe depression 15–19		6 (4%)		5 (7%)		0 (0%)		1 (4%)	
% Severe depression 20–27		9 (7%)		3 (4%)		2 (5%)		4 (17%)	
Depression (PHQ ≥ 10)	138		75		39		24		**0.002**
% Yes		50 (36%)		21 (28%)		14 (36%)		15 (63%)	
% No		88 (64%)		54 (72%)		25 (64%)		9 (37%)	
Moderately Severe or Severe Depression (PHQ ≥ 15)	138		75		39		24		0.29
% Yes		15 (11%)		8 (11%)		2 (5%)		5 (21%)	
% No		123 (89%)		67 (89%)		37 (95%)		19 (79%)	
PHQ-9 score	138	7.5 [3.0, 11.0]	75	7.0 [3.0, 11.0]	39	7.0 [4.0, 10.0]	24	11.0 [7.5, 14.0]	**0.005**
*Lupus QoL*									
Physical health	139	68.7 [32.5, 87.5]	75	65.6 [31.2, 84.3]	40	71.8 [48.4, 87.5]	24	56.2 [33.4, 85.9]	0.475
Pain	139	66.6 [41.6, 91.6]	75	58.3 [41.6, 83.3]	40	70.8 [41.6, 100.0]	24	66.6 [32.3, 91.6]	0.87
Planning	139	75.0 [37.5, 100.0]	75	75.0 [41.6, 100.0]	40	87.5 [25.0, 100.0]	24	62.5 [41.6, 100.0]	0.52
Intimacy	80	75.0 [37.5, 93.8]	38	68.8 [50.0, 87.5]	23	75.0 [37.5, 100.0]	19	50.0 [25.0, 75.0]	0.15
Burden to others	138	75.0 [50.0, 91.6]	75	75.0 [50.0, 91.6]	39	75.0 [41.6, 100.0]	24	75.0 [50.0, 91.6]	0.91
Emotional health	139	75.0 [50.0, 91.6]	75	75.0 [50.0, 91.6]	40	77.1 [54.1, 89.6]	24	70.8 [33.3, 83.3]	0.16
Body image	138	87.5 [60.0, 100.0]	74	91.6 [66.6, 100.0]	40	87.5 [62.5, 100.0]	24	65.6 [40.0, 95.8]	**0.021**
Fatigue	139	62.5 [43.7, 81.2]	75	68.7 [43.7, 87.5]	40	62.5 [50.0, 81.2]	24	46.9 [28.1, 59.4]	**0.005**
*Disease activity patterns*									
Chronic active disease	140	12 (9%)	75	3 (4%)	41	7 (17%)	24	2 (8%)	0.59
Relapsing-remitting	140	62 (44%)	75	30 (40%)	41	18 (44%)	24	14 (58%)	0.12
Long quiescence	140	53 (38%)	75	36 (48%)	41	13 (32%)	24	4 (17%)	**0.007**
Acute-new onset	140	13 (9%)	75	6 (8%)	41	3 (7%)	24	4 (17%)	0.25
**Activity index -SELENA-SLEDAI**	139	4.0 [0.0, 6.0]	75	4.0 [0.0, 6.0]	40	2.0 [0.0, 6.0]	24	2.5 [0.0, 4.0]	0.46
Active disease (SELENA-SLEDAI ≥ 4)	139	71 (51%)	75	42 (56%)	40	18 (45%)	24	11 (46%)	0.38
**Damage index - SLICC/ACR DI**	140	5.0 [4.0, 7.0]	75	5.0 [4.0, 7.0]	41	6.0 [4.0, 7.0]	24	5.0 [4.5, 6.0]	0.93
Damage (SLICC/ACR DI ≥ 1)	140	138 (99%)	75	75 (100%)	41	40 (98%)	24	23 (96%)	0.24
**Physician global assessment**	137	1.0 [1.0, 2.0]	74	1.0 [1.0, 2.0]	39	1.0 [0.0, 2.0]	24	1.0 [1.0, 2.0]	0.67
PhGA (high, scores 2 or 3)	137		74		39		24		0.40
% No		95 (69%)		53 (72%)		27 (69%)		15 (62%)	
% Yes		42 (31%)		21 (28%)		12 (31%)		9 (38%)	
**Patient Global Assessment**	139	5.0 [2.0, 6.0]	74	5.0 [2.0, 6.0]	41	4.0 [2.0, 5.0]	24	5.0 [3.0, 7.0]	0.24
PGA ≥ 7	139		74		41		24		0.29
% No		113 (81%)		60 (81%)		36 (88%)		17 (71%)	
% Yes		26 (19%)		14 (19%)		5 (12%)		7 (29%)	
**Self-rated health**	140		75		41		24		0.23
% Poor		11 (8%)		6 (8%)		2 (5%)		3 (12%)	
% Fair		51 (36%)		27 (36%)		13 (32%)		11 (46%)	
% Good		65 (47%)		32 (43%)		23 (56%)		10 (42%)	
% Excellent		13 (9%)		10 (13%)		3 (7%)		0 (0%)	
Poor health	140		75		41		24		0.22
% Yes		62 (44%)		33 (44%)		15 (37%)		14 (58%)	

*Lupus QoL* Lupus Quality of Life questionnaire, *PHQ* Patient Health Questionnaire, *SELENA-SLEDAI* Safety of Estrogens in Lupus Erythematosus National Assessment - Systemic Lupus Erythematosus Disease Activity Index, *SLICC/ACR DI* Systemic Lupus International Collaborating Clinics/American College of Rheumatology Damage, *PhGA* Physician global assessment

*Comparisons reported are between the low stress (Holmes–Rahe score < 150) and high stress (≥ 300) groups. Bold values indicate statistically significant results (p < 0.05)

Regarding SLE manifestations, patients in the high stress group more frequently had ophthalmologic (54.2% vs. 33.3%, *p* = 0.07), cutaneous (95.8% vs. 77.3%, *p* = 0.06) and cardiac (13% vs. 7%, *p* = 0.40) involvement, although these differences did not reach statistical significance. In terms of SLE-related comorbidities, patients with high stress levels were more likely to suffer from antiphospholipid syndrome (20.8% vs. 4%, *p* = 0.019). It should be noted that although more patients in the high stress level category experienced miscarriages, this difference did not reach statistical significance (39.1% vs. 24.1%, *p* = 0.18).

### Exposure to stressful life events, disease activity patterns, and patient beliefs

Regarding disease activity patterns, patients in the high stress group were less likely to experience long quiescence (17% vs. 48%, *p* = 0.007) and more likely to experience chronic active disease (8% vs. 4%, *p* = 0.59), although the latter did not reach statistical significance. Patients in the high stress group were also more likely to believe that stress is associated with disease onset (100% vs. 71.7%, *p* = 0.006) and disease flares (100% vs. 69%, *p* = 0.006).

### Multivariable logistic regression

To further evaluate the independent associations between stress exposure and clinical outcomes, a multivariable logistic regression analysis was performed (Table 4). The analysis showed that, compared to patients in the low stress group, patients in the high stress group were more likely to have antiphospholipid syndrome, (OR = 6.3, 95% CI 1.4–28.8; *p* = 0.017) and depression (OR = 4.3, 95% CI 1.6–11.3; *p* = 0.003) and less likely to have a long quiescent form of SLE (OR = 0.22, 95% CI 0.07–0.7; *p* = 0.010).

**Table 4:** Association between high stress exposurea and clinical outcomes in systemic lupus erythematosus using logistic regression analysis

	Unadjusted models			Models adjusted for gender and age at diagnosis		
	OR	95% CI	*p*-value	OR	95% CI	*p*-value
Antiphospholipid syndrome	6.32	(1.38, 28.82)	**0.017**	5.35	(1.17, 24.50)	**0.031**
Depression (PHQ ≥ 10)	4.29	(1.63, 11.28)	**0.003**	4.76	(1.75, 12.95)	**0.002**
Severe depression (PHQ ≥ 15)	2.20	(0.65, 7.53)	0.207	2.59	(0.70, 9.55)	0.153
Poor self-rated health	1.78	(0.70, 4.52)	0.224	1.92	(0.75, 4.96)	0.177
High PhGA	1.51	(0.58, 3.99)	0.401	1.73	(0.63, 4.75)	0.287
High disease activity (SELENA-SLEDAI ≥ 4)	0.67	(0.26, 1.67)	0.386	0.69	(0.27, 1.79)	0.448
Disease activity pattern: Long quiescence	0.22	(0.07, 0.70)	**0.010**	0.21	(0.07, 0.70)	**0.011**
PGA	1.77	(0.61, 5.07)	0.291	1.90	(0.64, 5.62)	0.249

*OR* odds ratio, *CI* confidence interval, *PHQ* Patient Health Questionnaire, *SELENA-SLEDAI* Safety of Estrogens in Lupus Erythematosus National Assessment - Systemic Lupus Erythematosus Disease Activity Index, *SLICC/ACR* *DI* Systemic Lupus International Collaborating Clinics/American College of Rheumatology Damage Index, *PhGA* Physician global assessment, *PGA* Patient global assessment, *BMI* body mass index

^a^High stress exposure defined as Holmes–Rahe score ≥ 300; reference group Holmes–Rahe score < 150. High stress exposure (≥ 300 LCU) was used as the independent variable in all models. Bold values indicate statistically significant results (p < 0.05)

## Discussion

This study quantitatively evaluates psychosocial stress exposure in the two years preceding SLE diagnosis and investigates its relationship with disease manifestations, activity patterns, and patient-reported outcomes. In this cross-sectional study, approximately half of patients with SLE were exposed to moderate to high levels of psychological stress in the two years preceding their SLE diagnosis. Elevated stress exposure was associated with a higher prevalence of comorbid conditions, including depression, fatigue, hypertension, obesity, and antiphospholipid syndrome, and was associated with a reduced probability of experiencing a long quiescent disease course. In addition, patients with higher stress exposure more frequently perceived stress as contributing to both disease onset and flares.

Our findings are consistent with a growing body of literature linking psychological stress to the course of autoimmune diseases. Epidemiologic studies have demonstrated an increased risk of autoimmune conditions, including SLE, among individuals with stress-related disorders, such as PTSD [24, 25]. Previous studies in SLE have largely relied on patient-reported associations between stress and disease flares [26], or on association of patient perceived stress with disease activity [14], without assessing stress exposure during a defined pre-diagnosis period or systematically examining its relationship with broader disease phenotype. Our study builds on this by quantitatively assessing stress exposure prior to diagnosis using a validated instrument and examining its association with disease phenotype and patient-reported outcomes.

The observed association between elevated stress exposure and patient-reported outcomes, including depression, fatigue, and body image is particularly relevant, as these manifestations strongly influence quality of life in SLE [27–29]. A longitudinal study showed that perceived stress plays an important role in SLE‑related fatigue, as decreases in stress were associated with meaningful reductions in fatigue [30]. A cross‑sectional study found that psychosocial stressors measured by the Holmes-Rahe Stress Scale Score were significantly associated with fatigue severity in SLE [31]. These findings suggest that fatigue in SLE is not solely driven by inflammatory disease activity, but is strongly influenced by stress, as observed in our cohort. Patients with higher stress exposure also reported significantly worse body image scores. Prior studies suggest that body image concerns may contribute to impaired quality of life in SLE and may reflect the combined burden of visible manifestations, treatment effects, and weight-related changes [29]. In our cohort, obesity was also more prevalent among patients with high stress exposure, which may have additionally influenced body image-related quality of life.

Beyond neuropsychiatric symptoms, higher stress exposure was associated with a greater prevalence of hypertension and obesity. Chronic psychological stress is known to promote activation of the sympathetic nervous system, increase catecholamine secretion, contributing to hypertension [32]. It has also been associated with maladaptive behaviors, such as poor dietary patterns which are associated with the development of obesity [32]. These factors may contribute to cardiometabolic risk, which is already elevated in SLE [33]. Although causality cannot be established, and these associations may reflect shared underlying risk factors, behavioral influences, or non-causal relationships, our findings suggest that stress exposure preceding diagnosis may identify a subgroup of patients with SLE at an increased risk for cardiovascular comorbidities.

A key finding in this study was the association between high levels of stress exposure and antiphospholipid syndrome. Duca et al. in a cross-sectional analysis involving patients with clinically inactive SLE, found that increased depression and anxiety were significantly associated with the presence of antiphospholipid antibodies, including anticardiolipin, anti-β2 glycoprotein I, and lupus anticoagulant, as well as with biomarkers related to impaired fibrinolysis [34]. Overall, these results suggest that experiencing stress before illness onset could be related to prothrombotic immune characteristics in individuals with SLE. However, because the timing of antiphospholipid syndrome diagnosis was not recorded in our cohort, antiphospholipid syndrome may have preceded SLE diagnosis in some patients and contributed to the exposure to stressful life events in the premorbid period, potentially introducing confounding. Moreover, this finding should be interpreted with caution given the small number of events and the width of the reported confidence intervals.

Stress exposure was also associated with disease activity patterns. Patients with higher stress levels were less likely to experience a long quiescent disease course. Prior studies have suggested that stress may precipitate disease flares in SLE, primarily based on patient-reported experiences or associations between perceived stress and symptom exacerbation [14, 26, 35]. Our findings extend this by demonstrating an association between quantified stress exposure pre-diagnosis and ongoing disease patterns. Patients with higher stress exposure were also more likely to believe that stress contributed to both disease onset and flares. Although these perceptions are not evidence of causation, they highlight the importance patients place on stress in relation to their disease and daily lives.

Stress-related activation of neuroendocrine pathways may help explain the associations observed in our study. Chronic psychological stress activates neuroendocrine stress pathways, primarily the hypothalamic-pituitary-adrenal axis (HPA) and the sympathetic nervous system, resulting in alterations in cortisol and catecholamine signaling that influence immune regulation [5]. While acute activation of these systems exerts largely anti-inflammatory effects, prolonged stress exposure is associated with maladaptive neuroendocrine responses, including impaired HPA axis feedback and reduced glucocorticoid receptor sensitivity [36]. This dysregulation diminishes the immunomodulatory effects of cortisol and is associated with sustained pro-inflammatory cytokine signaling and impaired immune tolerance [36]. In SLE, experimental and clinical evidence suggests that such stress-related neuroendocrine-immune dysfunction provides a biologically plausible mechanism linking psychological stress to immune activation, autoantibody production, and persistent inflammatory activity [14, 34, 36, 37].

These findings have important clinical implications. Psychological stress exposure preceding SLE diagnosis may help identify a subset of patients at an increased risk for comorbidities, including depression, cardiometabolic disease, and antiphospholipid syndrome, as well as for less favorable disease activity patterns. Although causality cannot be inferred, inquiring about recent life stressors at the time of diagnosis may assist with early risk stratification and patient counseling. Collectively, these findings support a more holistic approach to early SLE management that integrates psychosocial risk assessment alongside traditional clinical and serologic evaluation.

Our study has several limitations. Stress exposure was assessed retrospectively and may be subject to recall bias, which may have influenced the accuracy and timing of reported life events. The use of a structured and widely used instrument focusing on major life events may help mitigate this limitation, although it does not eliminate it. The study was conducted at a single tertiary care center, limiting generalizability, whereas the modest sample size may have limited power to detect associations with less frequent outcomes. The relatively small size of the high stress subgroup may have further limited statistical power and the stability of regression estimates. Furthermore, the cross-sectional design limits the ability to establish temporal relationships and precludes causal inferences between stress exposure and disease expression. In addition, the timing of comorbidity onset relative to stress exposure was not captured, further limiting causal interpretation of these associations. Residual confounding from unmeasured variables, including disease duration, treatment exposure, and socioeconomic factors, cannot be excluded. In addition, the inclusion of patients with overlapping autoimmune conditions may introduce heterogeneity and could have influenced the observed associations; however, exclusion of these patients was not performed due to sample size constraints. As data were not collected prospectively, we could not evaluate whether psychological stress has any association with SLE flares. Finally, while the Holmes-Rahe scale captures objective life events providing a standardized measurement of the stress induced by life events, it does not assess individual stress perception, coping strategies, or resilience, which may modify the impact of stress on disease expression. Additionally, we employed the original 1967 version of the scale, which includes items that may be outdated or inconsistent with modern social norms. A revised version addressing these limitations was published in 2023 [22], but was not available during the conduct of our study. Thus, future studies would benefit from incorporating measures of perceived stress alongside stressful life event scales.

## Conclusion

In summary, this study demonstrates that increased psychosocial stress exposure during the two years prior to SLE diagnosis is associated with a higher prevalence of comorbidities, less favorable patterns of disease activity, and greater patient-reported symptom burden. These findings highlight the clinical importance of psychological stress in SLE management. Further research is needed to determine whether interventions targeting stress may positively affect disease trajectory and patient-centered outcomes.

## Data Availability

The data that support the findings of this study are not openly available due to reasons of sensitivity and are available from the corresponding author upon reasonable request.

## Declarations

### Use of Artificial Intelligence

The authors used artificial intelligence tools to assist with language editing and manuscript refinement. All content was reviewed, verified, and approved by the authors, who take full responsibility for the accuracy and integrity of the manuscript.
